# High incidence of extensive chronic graft-versus-host disease in patients with the *REG3A* rs7588571 non-GG genotype

**DOI:** 10.1371/journal.pone.0185213

**Published:** 2017-09-25

**Authors:** Daisuke Koyama, Makoto Murata, Ryo Hanajiri, Shingo Okuno, Sonoko Kamoshita, Jakrawadee Julamanee, Erina Takagi, Daiki Hirano, Kotaro Miyao, Reona Sakemura, Tatsunori Goto, Fumihiko Hayakawa, Aika Seto, Yukiyasu Ozawa, Koichi Miyamura, Seitaro Terakura, Tetsuya Nishida, Hitoshi Kiyoi

**Affiliations:** 1 Department of Hematology and Oncology, Nagoya University Graduate School of Medicine, Nagoya, Aichi, Japan; 2 Department of Hematology, Japanese Red Cross Nagoya First Hospital, Nagoya, Aichi, Japan; University of Kentucky, UNITED STATES

## Abstract

Regenerating islet-derived protein 3 alpha (REG3A) is a biomarker of lower gastrointestinal graft-versus-host disease (GVHD); however, the biological role of REG3A in the pathophysiology of GVHD is not understood. Here, we examined the association between a single nucleotide polymorphism in the *REG3A* gene, rs7588571, which is located upstream and within 2 kb of the *REG3A* gene, and transplant outcomes including the incidence of GVHD. The study population consisted of 126 adult Japanese patients who had undergone bone marrow transplantation from a HLA-matched sibling. There was no association between rs7588571 polymorphism and the incidence of acute GVHD. However, a significantly higher incidence of extensive chronic GVHD was observed in patients with the rs7588571 non-GG genotype than in those with the GG genotype (Odds ratio 2.6; 95% confidence interval, 1.1–6.0; P = 0.029). Semi-quantitative reverse transcription PCR demonstrated that the rs7588571 non-GG genotype exhibited a significantly lower *REG3A* mRNA expression level than the GG genotype (P = 0.032), and Western blot analysis demonstrated that the rs7588571 non-GG genotype exhibited a trend toward lower REG3A protein expression level than the GG genotype (P = 0.053). Since REG proteins have several activities that function to control intestinal microbiota, and since intestinal dysbiosis is in part responsible for the development of GVHD, our findings lead to the novel concept that REG3A could have some protective effect in the pathogenesis of GVHD through the regulation of gut microbiota.

## Introduction

Graft-versus-host disease (GVHD) is a major complication after allogeneic hematopoietic stem cell transplantation [1]. Several plasma biomarkers have been identified as promising tools for identification of patients at higher risk for GVHD morbidity, treatment unresponsiveness, and mortality after GVHD [2–6]. Regenerating islet-derived 3-alpha (REG3A) is a biomarker of gastrointestinal GVHD, and higher plasma concentration of REG3A is associated with higher non-relapse mortality (NRM) [4]. REG3A is a protein that is secreted into the intestinal mucosa by Paneth cells and intestinal epithelial cells [7], and therefore the elevation of plasma REG3A concentration in GVHD is assumed to be a result of its leakage to the systemic circulation due to disruption of the intestinal epithelial barrier [4].

Currently, there is no direct evidence for a biological role for REG3A in the pathophysiology of GVHD. However, REGIIIγ, a mouse homologue of human REG3A, has several activities that function to control intestinal microbiota [8, 9], and intestinal dysbiosis is in part responsible for the development of GVHD [10]. These phenomena suggest that REG3A may play some role in GVHD pathogenesis. Although it is not clear whether the *REG3A* gene has a functional variant, we focused on a single nucleotide polymorphism (SNP), rs7588571, which is located upstream and within 2 kb of the *REG3A* gene, in the putative promoter region of *REG3A* (http://www.genecards.org/). We hypothesized that the SNP rs7588571 might have an effect on the incidence of GVHD or other transplant outcomes as a consequence of differences in the expression level of REG3A.

## Patients and methods

### Patients

A total of 126 adult Japanese patients were selected according to the following inclusion criteria: (1) the graft was bone marrow; (2) the donor was HLA-identical sibling; (3) short-term methotrexate and cyclosporine were given as GVHD prophylaxis; (4) genomic DNA samples and clinical data were available. Informed consent was obtained from all patients. The study was approved by the ethics committees at the Nagoya University Hospital and the Japanese Red Cross Nagoya First Hospital.

### Definitions

High risk diseases included acute myeloid leukemia beyond second complete remission, acute lymphoblastic leukemia without Philadelphia chromosome beyond first complete remission, acute lymphoblastic leukemia with Philadelphia chromosome, chronic myeloid leukemia in the accelerated phase or blastic crisis, myelodysplastic syndrome with excess of blasts, chronic myelomonocytic leukemia, malignant lymphoma, and multiple myeloma. Standard risk diseases included all other diseases. The conditioning regimen was classified as myeloablative conditioning or reduced intensity conditioning according to established criteria [11].

### rs7588571 genotyping

Genomic DNA was extracted from a patient’s peripheral blood or bone marrow with the QIAamp DNA Blood Mini Kit (QIAGEN sciences, Germantown, MD, USA). Genotyping was performed using TaqMan SNP Genotyping Assays (Assay ID: C__32401015_10, Applied Biosystems, Foster City, California, USA) on ABI 7300 Real-Time PCR systems (Applied Biosystems).

### Semi-quantitative reverse transcription PCR for *REG3A*

Three B-lymphoblastoid cell lines (B-LCLs) per each rs7588571 genotype were chosen from previously established B-LCLs [12]. Total RNA was extracted from B-LCLs and Panc-1 cell line cells which is a pancreatic cancer cell line expressing *REG3A* using the RNeasy Mini kit (Qiagen, Manchester, UK), and 5 μg of total RNA was reverse transcribed using the Superscript III First-Strand Synthesis System for RT-PCR (Invitrogen, Carlsbad, CA, USA). The resulting cDNA, which was equivalent to 750 ng of total RNA, was PCR amplified under the following PCR settings using Takara Ex Taq (TakaraBio, Shiga, Japan) with TaqMan^®^ Gene Expression Assays (assay ID: *REG3A*, Hs00170171_m1; *GAPDH* as the internal control, Hs99999905_m1; Applied Biosystems): initial denaturation at 98°C for 30 sec; 50 cycles of 98°C for 10 sec and 60°C for 60 sec; final extension at 72°C for 7 min. The PCR product was electrophoresed in a 4% agarose gel and stained with ethidium bromide. The intensity of each PCR band was quantified using ImageJ software (version 1.50i, NIH), and the expression of *REG3A* adjusted by *GAPDH* expression was evaluated.

### Western blot analysis for REG3A protein

Western blot analysis was performed as previously described [13]. Briefly, the whole-cell lysates were prepared from LCLs and Panc-1 cell line cells, separated by SDS-PAGE, and transferred onto the Immun-Blot PVDF Membrane (BIO-RAD, Tokyo, Japan). Anti-Reg3a antibody (Abcam, MA, USA), β-Actin (13E5) Rabbit mAb (Cell Signaling Technology, MA, USA), and HRP-conjugated anti-rabbit IgG (GE Healthcare, Buckinghamshire, England) were used in accordance with the manufacturer’s instruction. Proteins were visualized with the ECL-prime Western Blotting Detection System (GE Healthcare) using the ImageQuant LAS-4000 mini (FUJIFILM, Tokyo, Japan). The β-actin was used as a loading control.

### Statistical analysis

Overall survival (OS) was calculated by the Kaplan-Meier method [14]. NRM was defined as mortality due to any cause other than relapse or disease progression. Cumulative incidences of NRM and relapse were estimated using Gray’s method, with relapse and NRM, respectively, as a competing risk [15]. Acute GVHD was graded by established criteria [16]. Chronic GVHD was evaluated in patients who survived beyond day 100, and was classified as limited or extensive type [17]. The chi-square test was used for categorical variables, and the Mann-Whitney U test was used for continuous variables. A multiple logistic regression model was used to identify risk factors associated with acute and chronic GVHD [18]. The Cox proportional hazards model was used for multivariate analyses of OS [19], while the Fine and Gray proportional hazards model was used for NRM, and relapse [20]. The following variables were evaluated: patient age (≤35 vs. >35); donor-recipient gender combination (female to male vs. others); disease (standard risk disease vs. high risk disease); conditioning regimen (myeloablative regimen vs. reduced-intensity regimen); recipient cytomegalovirus serostatus (positive vs. negative); years of transplantation (1987–1996 vs. 1997–2006); and rs7588571 genotype (GG genotype vs. non-GG genotype). The history of acute GVHD was included as a variable in the chronic GVHD analysis. Variables with a P-value <0.1 in the univariate analysis were entered into the stepwise selection method. All tests were two-sided, and a P-value <0.05 was considered significant.

## Results

### Frequencies of rs7588571 genotypes

The frequency of patients with GG, AG, and AA genotypes of the *REG3A* rs7588571 was 40.5% (n = 51), 43.7% (n = 55), and 15.9% (n = 20), respectively, and these frequencies were compatible with those reported in the HapMap-JPT database (GG genotype, 30.2%; AG genotype, 57.0%; and AA genotype, 12.8%).

### Patient characteristics categorized by rs7588571 genotype

Patient characteristics were categorized by the rs7588571 genotype (Table 1). There was no significantly different patient characteristic among the three rs7588571 genotypes.

**Table 1:** Patient characteristics categorized by rs7588571 genotype.

	Genotype			P-value
	GG	AG	AA	
**Number of patients**	51	55	20	
**Median age (range), y**	35 (15–55)	37 (16–56)	38 (17–61)	0.57
**Gender, n (%)**				0.55
Male	27 (53)	34 (62)	10 (50)	
Female	24 (47)	21 (38)	10 (50)	
**Gender mismatch between patient and donor, n (%)**				0.34
Female to male	12 (24)	19 (35)	4 (20)	
Other combinations	39 (76)	36 (65)	16 (80)	
**Diagnosis, n (%)**				0.97
AML	16 (30)	15 (27)	5 (25)	
ALL	10 (20)	9 (16)	4 (20)	
CML	11 (22)	16 (29)	8 (40)	
MDS/CMMoL	5 (10)	7 (13)	1 (5)	
Other malignancies	1 (2)	1 (2)	0 (0)	
AA/PNH	8 (16)	7 (13)	2 (10)	
**Disease risk, n (%)**				0.18
Standard	39 (76)	38 (69)	18 (90)	
High	12 (24)	17 (31)	2 (10)	
**Conditioning, n (%)**				1
Myeloablative regimen	49 (96)	53 (96)	19 (95)	
Reduced-intensity regimen	2 (4)	2 (4)	1 (5)	
**GVHD prophylaxis, n (%)**				1
sMTX + CsA	51 (100)	55 (100)	20 (100)	
**Graft source, n (%)**				1
Bone marrow	51 (100)	55 (100)	20 (100)	
**Donor type, n (%)**				1
HLA-matched sibling donor	51 (100)	55 (100)	20 (100)	
**Cytomegalovirus serostatus, n(%)**				0.85
Positive	42 (82)	42 (77)	14 (70)	
Negative	8 (16)	9 (16)	4 (20)	
Unknown	1 (2)	4 (7)	2 (10)	
**Year of transplant, n (%)**				0.57
1987–1996	30 (59)	28 (51)	9 (45)	
1997–2006	21 (41)	27 (49)	11 (55)	

AML indicates acute myeloid leukemia; ALL, acute lymphoblastic leukemia; CML, chronic myeloid leukemia; MDS, myelodysplastiv syndrome; CMMoL, chronic myelomonocytic leukemia, AA, aplastic anemia; PNH, paroxysmal nocturnal hemoglobinuria; sMTX, short-term methotrexate; CsA, cyclosporine.

### Effect of rs7588571 genotype on acute GVHD

Of 125 evaluable patients, grade I–IV, grade II–IV, skin, liver and gut acute GVHD developed in 51 (41%), 19 (15%), 47 (38%), 3 (2%) and 9 (7%) patients, respectively (Table 2). There was no significant association between rs7588571 genotype and the incidence of each acute GVHD group (Table 2). Multivariate analysis demonstrated that higher patient age (>35) was a risk factor for grade II–IV acute GVHD (Odds ratio, 4.9; 95% confidence interval [CI], 1.5–15.7; P = 0.007).

**Table 2:** Incidence of GVHD in each rs7588571 genotype.

	Acute GVHD					Chronic GVHD	
	Grade I-IV	Grade II-IV	Skin involvement	Liver involvement	Gut involvement	Limited and Extensive	Extensive
	(n = 51)	(n = 19)	(n = 47)	(n = 3)	(n = 9)	(n = 56)	(n = 45)
**Total, n (%)**	51/125 (41)	19/125 (15)	47/125 (38)	3/125 (2)	9/125 (7)	56/119 (47)	45/119 (38)
**GG, n (%)**	18/51 (35)	7/51 (14)	17/51 (33)	0/51 (0)	1/51 (2)	21/50 (42)	13/50 (26)
**AG, n (%)**	25/54 (46)	9/54 (17)	23/54 (43)	3/54 (6)	6/54 (11)	26/52 (50)	23/52 (44)
**AA, n (%)**	8/20 (40)	3/20 (15)	7/20 (35)	0/20 (0)	2/20 (10)	9/17 (53)	9/17 (53)
**P-value**	0.51	0.94	0.60	0.21	0.15	0.66	0.063
**GG, n (%)**	18/51 (35)	7/51 (14)	17/51 (33)	0/51 (0)	1/51 (2)	21/50 (42)	13/50 (26)
**non-GG (AG and AA), n (%)**	33/74 (45)	12/74 (16)	30/74 (41)	3/74 (4)	8/74 (11)	35/69 (51)	32/69 (46)
**P-value**	0.36	0.80	0.46	0.27	0.081	0.36	0.035

### Effect of rs7588571 genotype on chronic GVHD

Of 119 evaluable patients, limited and extensive chronic GVHD developed in 56 (47%) patients (Table 2). There was no significant association between rs7588571 genotype and the incidence of limited and extensive chronic GVHD (Table 2). Multivariate analysis demonstrated that female to male transplant and high-risk disease were risk factors for limited and extensive chronic GVHD (Odds ratio, 4.0; 95% CI, 1.6–10.0; P = 0.0031 for female to male transplant; 4.2, 1.6–11.4, P = 0.0045 for high risk disease).

Extensive chronic GVHD developed in 45 (38%) of 119 evaluable patients (Table 2). The incidence of extensive chronic GVHD in patients with the GG, AG and AA genotype was 26%, 44% and 53%, respectively (P = 0.063). The incidence of extensive chronic GVHD was significantly higher in patients with the non-GG genotype than in patients with the GG genotype (46% vs. 26%, P = 0.035). Multivariate analysis demonstrated that the non-GG genotype was a risk factor for higher incidence of extensive chronic GVHD (Odds ratio, 2.6; 95% CI, 1.1–6.0; P = 0.029) (Table 3). Female to male transplantation and high-risk disease were also significant risk factors. We performed an additional analysis on the group of patients without acute GVHD. The *REG3A* rs7588571 non-GG genotype showed a trend toward a higher incidence of extensive chronic GVHD than the GG genotype (45% vs. 24%, P = 0.087).

**Table 3:** Univariate and multivariate analyses of risk factors for extensive chronic GVHD.

Risk factors	Univariate		Multivariate	
	Odds ratio (95% CI)	P-value	Odds ratio (95% CI)	P-value
**Gender mismatch**				
Others	1		1	
Female to Male	3.4 (1.5−8.0)	0.0041	3.2 (1.3−7.8)	0.0099
**Disease risk**				
Standard	1		1	
High	3.2 (1.3−7.6)	0.011	3.0 (1.2−7.8)	0.020
**rs7588571**				
GG	1		1	
non-GG	2.5 (1.1−5.4)	0.025	2.6 (1.1−6.0)	0.029

CI indicates confidence interval.

Covariates used were age, donor–recipient gender combination, disease risk, conditioning, recipient cytomegalovirus serostatus, history of acute GVHD, year of transplantation and genotype of rs7588571.

### Effect of rs7588571 genotype on other transplant outcomes

In 109 evaluable patients, the relapse rate at 4-years after transplantation was 23.5%. The rs7588571 genotype did not affect the relapse rate, and high-risk disease was the only significant factor for higher relapse rate (hazard ratio [HR], 3.0; 95% CI, 1.3–7.1; P = 0.012).

In 109 evaluable patients, NRM at 4-years after transplantation was 16.9%. The rs7588571 genotype did not affect NRM, and there was no significant factor for higher NRM.

In 126 evaluable patients, OS at 4-years after transplantation was 62.8%. The rs7588571 genotype did not affect OS, and high-risk disease was the only significant factor for lower OS (HR, 0.36; 95% CI, 0.19–0.69; P = 0.0019).

### Comparison of the expression level of *REG3A* mRNA in cells of each rs7588571 genotype

**Fig 1:**
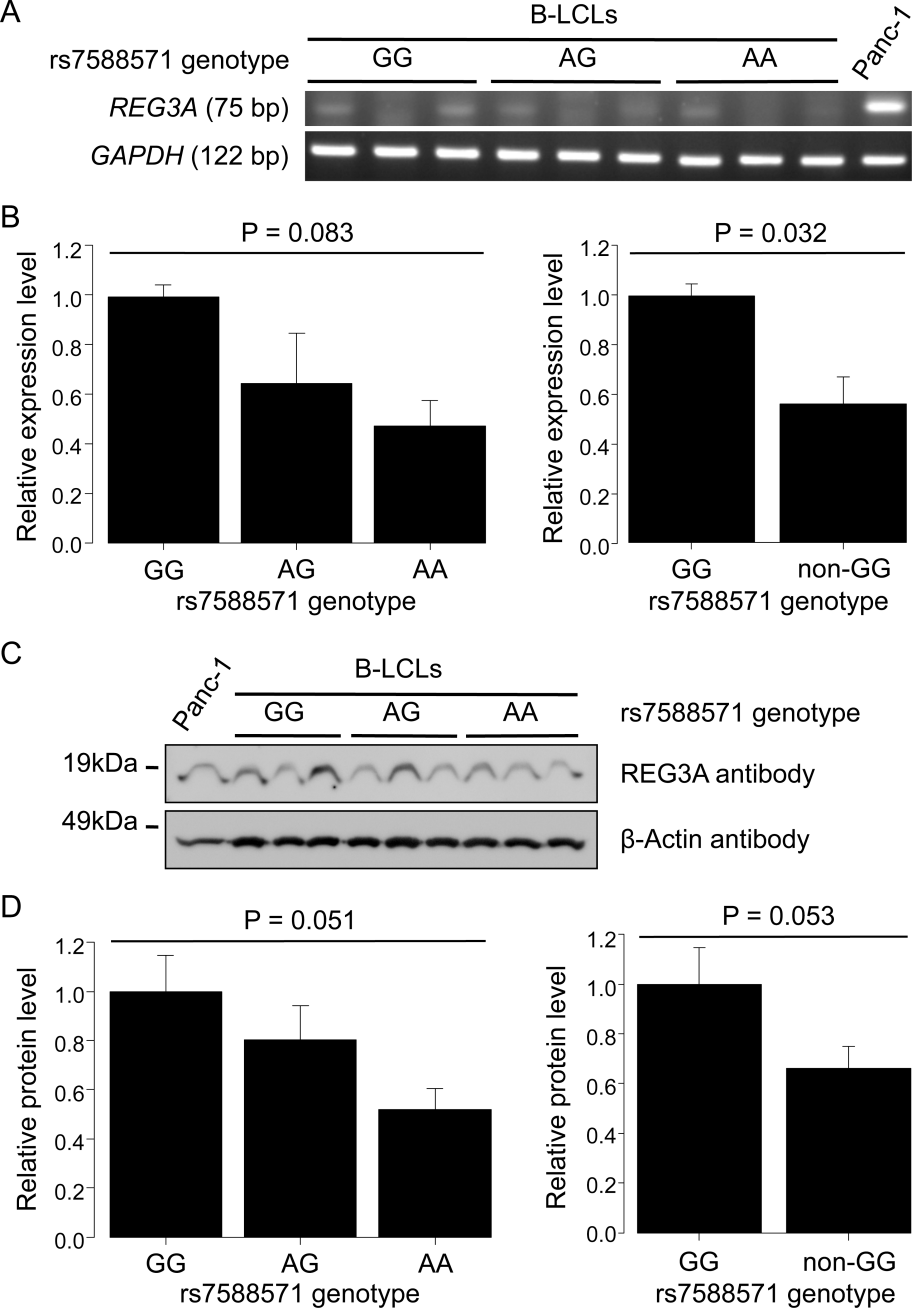
Expression level of *REG3A* in B-LCLs of each *REG3A* rs7588571 genotype. **A. Semi-quantitative reverse transcription PCR for *REG3A***. Total RNA was extracted from three B-LCLs for each genotype and reverse transcribed. The cDNA equivalent to the same amount of total RNA was subjected to reverse trancription PCR. Panc-1, which is a pancreatic cancer cell line expressing *REG3A*, was used as a positive control. *GAPDH* was used as an internal control. **B. Comparison of *REG3A* expression level among rs7588571 genotypes**. The intensity of each PCR band was quantified, and expression levels of *REG3A* were normalized to *GAPDH* expression levels. The normalized expression level of *REG3A* in the GG genotype was used as a reference. The relative expression levels of *REG3A* were compared among the three rs7588571 genotypes using one-way ANOVA (left) and between the GG and non-GG genotypes using a t-test (right). Data of two independent experiments (mean ± S.E.M.) each performed in triplicate are shown. **C. Western blot for REG3A protein**. Lysate was extracted from three B-LCLs for each genotype that were used in Fig 1A. The same amount of lysate was applied in SDS-PAGE. Panc-1 was used as a positive control. The β-actin was used as a loading control. **D. Comparison of expression level of REG3A protein among rs7588571 genotypes**. The expression level of REG3A protein was quantified. The expression level of REG3A protein in the GG genotype was used as a reference. The relative expression levels of REG3A protein were compared among the three rs7588571 genotypes using one-way ANOVA (left) and between the GG and non-GG genotypes using a t-test (right). Data of two independent experiments (mean ± S.E.M.) are shown.

The rs7588571 is located upstream and within 2 kb of the *REG3A* gene, and lies in the putative promoter region of *REG3A* (http://www.genecards.org/). We therefore investigated the expression of *REG3A* mRNA in cells of each rs7588571 genotype. However, due to the difficulty in collecting human intestinal tissue samples of each rs7588571 genotype, preferably at least three samples per each genotype for triplicate experiments, and due to the failure to detect *REG3A* mRNA in peripheral blood mononuclear cells using real-time PCR (data not shown), we needed to find alternative cells for each rs7588571 genotype that had a common human tissue origin. Therefore, three B-LCLs per each rs7588571 genotype were chosen from previously established B-LCLs [12] and used as alternative materials for semi-quantitative reverse transcription PCR for *REG3A* expression analysis (Fig 1A). The relative expression level of *REG3A* varied among rs7588571 genotypes (P = 0.083) (Fig 1B, left). The relative expression level of the *REG3A* rs7588571 non-GG genotype was significantly lower than that of the GG genotype (P = 0.032) (Fig 1B, right).

### Comparison of the expression level of REG3A protein in cells of each rs7588571 genotype

We next investigated the expression of REG3A protein in three B-LCLs per each rs7588571 genotype (Fig 1C). The relative expression level of REG3A protein varied among rs7588571 genotypes (P = 0.051) (Fig 1D, left). The *REG3A* rs7588571 non-GG genotype showed a trend toward a lower REG3A protein level than the GG genotype (P = 0.053) (Fig 1D, right).

## Discussion

To date, there have been few reports supporting the role of REG3A in the pathophysiology of GVHD. In murine GVHD models, REGIIIγ, a mouse homologue of human REG3A, was upregulated in villous enterocytes [21]. Host deficiency of IL-22, which is a cytokine upstream of REG3A [22], leads to an increase in gastrointestinal GVHD [23]. In humans, it was reported that the expression of IL-22 in peripheral blood mononuclear cells was elevated in pediatric patients who had active chronic GVHD [24].

In the present study, the rs7588571 non-GG genotype was associated with higher incidence of extensive chronic GVHD after bone marrow transplantation (BMT) from a HLA-matched sibling than the GG genotype. Additionally, the rs7588571 non-GG genotype exhibited lower expression levels of *REG3A* mRNA (P = 0.032) and REG3A protein (P = 0.053) than the GG genotype. REG proteins have several activities that function to control gut microbiota, such as bactericidal activities towards gram-positive bacteria and maintenance of spatial segregation between microbiota and the host [8, 9]. Gut microbiota supply metabolites including short chain fatty acids as an intestinal energy source [25]. Gut microbiota also shape the mucosal immune system by regulating the differentiation and expansion of immuno-regulatory cells, including regulatory T cells [26–29]. Thus, gut and its microbiota influence each other to maintain intestinal homeostasis. Recent studies have revealed the relationship between intestinal dysbiosis and the development of intestinal GVHD [10, 30–35] and found that regulatory T cells are one of the key players in controlling graft-versus-host response [36]. Based on these combined data, our findings lead to the novel concept that REG3A could have some protective effect in the pathogenesis of GVHD through the regulation of gut microbiota.

Nonetheless, the non-GG genotype was a risk factor for extensive chronic GVHD, but not for gut acute GVHD (Table 2). Several murine studies have suggested that primed T cells can initiate GVHD at sites other than their original priming sites [37, 38]. In the field of autoimmunity, changes in gut microbiota have been proposed to account for the development of autoimmune diseases, such as rheumatoid arthritis and systemic sclerosis, in which organs other than the gastrointestinal tract are targeted [39]. We also previously reported that an SNP in the *CCR9* gene, which is involved in homing of T cells to the small intestine, was associated with the incidence of acute and chronic skin GVHD [40]. These findings are consistent with the participation of T cells that are primed at gut-associated lymphoid tissue in the induction of acute and chronic GVHD in other sites.

There are some limitations to our study. First, we selected patients who had undergone HLA-matched sibling BMT with uniform GVHD prophylaxis. This patient selection contributed to the formation of a homogenous study cohort and to the performance of analyses without consideration of HLA incompatibility, stem cell source or transplant procedure biases. However, by using this small study cohort other important factors might have been missed, as discussed in another report [41]. Conversely, as discussed in a recent paper [42], most, but not necessarily all, of the results in SNP studies evaluating between 50 and 350 individuals may be false-positives. Thus, a verification using a cohort of much larger number of patients than this study is essential. Second, the NIH criteria for chronic GVHD was not applied in this study because the present cohort included many patients who had received transplantation before the widespread use of NIH grading system in Japan. The study cohort of patients who had diagnosis and staging of chronic GVHD based on the NIH criteria may enable us to perform more precise and comprehensive analysis of the association between REG3A polymorphism and chronic GVHD. Third, we demonstrated differences in the expression levels of *REG3A* mRNA and REG3A protein by rs7588571 genotypes in B-LCLs. However, those in intestinal tissue have not yet been determined. In addition, the difference in the degree of dysbiosis in post-transplant patients with each rs7588571 genotype should also be investigated.

In summary, rs7588571 genotype was associated with the expression level of *REG3A* mRNA and REG3A protein and the incidence of extensive chronic GVHD in patients who had received an HLA-matched sibling bone marrow transplant. REG3A may play some role in the pathophysiology of GVHD in addition to being a GVHD biomarker. A validation study in a large cohort is needed to confirm the impact of *REG3A* polymorphism on transplant outcome, and future in vivo studies will contribute to clarifying the exact role of REG3A in GVHD.
